# Supplementary material for: Exome sequencing identifies rare damaging variants in ATP8B4 and ABCA1 as risk factors for Alzheimer’s disease
Source: Nat Genet. 2022 Nov 21;54(12):1786–94. doi: 10.1038/s41588-022-01208-7 (PMC9729101; doi:10.1038/s41588-022-01208-7)
Supplement: Supplementary file 2 — Reporting Summary [file 41588_2022_1208_MOESM2_ESM.pdf]

nature portfolio

Corresponding author(s): Henne Holstege
Marc Hulsman
Gaël Nicolas
Jean Charles Lambert

Last updated by author(s): Jul 29, 2022

Reporting Summary

Nature Portfolio wishes to improve the reproducibility of the work that we publish. This form provides structure for consistency and transparency in reporting. For further information on Nature Portfolio policies, see our Editorial Policies and the Editorial Policy Checklist.

Statistics

For all statistical analyses, confirm that the following items are present in the figure legend, table legend, main text, or Methods section.

n/a	Confirmed	
☐	☒	The exact sample size (*n*) for each experimental group/condition, given as a discrete number and unit of measurement
☐	☒	A statement on whether measurements were taken from distinct samples or whether the same sample was measured repeatedly
☐	☒	The statistical test(s) used AND whether they are one- or two-sided<br>*Only common tests should be described solely by name; describe more complex techniques in the Methods section.*
☐	☒	A description of all covariates tested
☐	☒	A description of any assumptions or corrections, such as tests of normality and adjustment for multiple comparisons
☐	☒	A full description of the statistical parameters including central tendency (e.g. means) or other basic estimates (e.g. regression coefficient) AND variation (e.g. standard deviation) or associated estimates of uncertainty (e.g. confidence intervals)
☐	☒	For null hypothesis testing, the test statistic (e.g. F, t, r) with confidence intervals, effect sizes, degrees of freedom and P value noted<br>*Give P values as exact values whenever suitable.*
☒	☐	For Bayesian analysis, information on the choice of priors and Markov chain Monte Carlo settings
☒	☐	For hierarchical and complex designs, identification of the appropriate level for tests and full reporting of outcomes
☐	☒	Estimates of effect sizes (e.g. Cohen's d, Pearson's r), indicating how they were calculated

Our web collection on statistics for biologists contains articles on many of the points above.

Software and code

Policy information about availability of computer code

Data collection	No software was used.
Data analysis	BCFtools (version 1.8)<br>Bedtools (version 2.26.0)<br>BWA (version 0.7.15-r1140)<br>EAGLE (version 2.4)<br>GATK (version 3.8-1): BQSR, HaplotypeCaller, VQSR, CombineGVCFs, GenotypeGVCFs<br>LOFTEE (version 1.0.2)<br>MACS (version 1.4)<br>Matplotlib (version 3.1.1)<br>Numpy (version 1.17.4)<br>Picard tools (version 2.10.5)<br>Python (version 2.7, 3.6)<br>R (version 3.4.3)<br>R-MASS package (version 7.3-51.5)<br>R-lmtest package (version 0.9-35)<br>VEP (version 94.5)<br>VerifyBamID2 (version 1.0.5)<br>Samblaster (version 0.1.24)<br>Samtools (version 1.8)<br>Scipy (version 1.4.1)<br>Seekin (version 1.0)<br>SKLearn (version 0.20.3)

Custom scripts: https://github.com/holstegelab/shortread_seq_analysis
https://doi.org/10.5281/zenodo.6827458

For manuscripts utilizing custom algorithms or software that are central to the research but not yet described in published literature, software must be made available to editors and reviewers. We strongly encourage code deposition in a community repository (e.g. GitHub). See the Nature Portfolio guidelines for submitting code & software for further information.

Data

Policy information about availability of data

All manuscripts must include a data availability statement. This statement should provide the following information, where applicable:

- Accession codes, unique identifiers, or web links for publicly available datasets
- A description of any restrictions on data availability
- For clinical datasets or third party data, please ensure that the statement adheres to our policy

The genetic variants analyzed during this study are listed in the Supplementary Data attached to this Letter.
Summary statistics of the discovery analysis were deposited to the Zenodo Digital Archive: 10.5281/zenodo.6818051
The ADSP dataset (which includes the ADNI dataset) used in this analysis is publicly available upon request: https://dss.niagads.org/datasets/
Accession numbers of data used in this analysis:
ADSP DBGap: phs000572.v7.p4 (stage-1)
ADSP Niagads: ng00067.v2 (stage-2)

Field-specific reporting

Please select the one below that is the best fit for your research. If you are not sure, read the appropriate sections before making your selection.

☒ Life sciences ☐ Behavioural & social sciences ☐ Ecological, evolutionary & environmental sciences

For a reference copy of the document with all sections, see nature.com/documents/nr-reporting-summary-flat.pdf

Life sciences study design

All studies must disclose on these points even when the disclosure is negative.

Sample size	Raw data used in this study was collected by the ADES consortium, the ADSP consortium, the ADNI consortium, and researchers who collected the StEP-AD, Knight-ADRC, UCSF cohorts. Sample size was not pre-determined and was chosen based on all known available cohorts with relevant data collected to date, after quality control steps were performed in each cohort (described in detail in Supplementary Information) in particular to avoid any sample duplications and family relations. The sample size was calculated as the number of individuals summed across all studies in the meta-analysis, N=32,558.
Data exclusions	We excluded samples and variants from the analysis based on extensive quality control procedures as thoroughly explained in the Methods and Supplemental Note. Please see the Supplemental Note, Supplementary Tables 3, 4, and 5, on the effect on quality control measures taken (sample QC and variant QC).
Replication	After sample QC, we first compared gene-based rare-variant burdens between 12,652 AD cases and 8,693 controls in a Stage-1 analysis. To confirm burden signals identified in Stage 1, we applied an analysis model consistent with Stage-1 to an independent Stage-2 dataset, which after QC, comprised 3,384 cases and 7,829 controls. This confirmed the AD-association of rare damaging variants in the SORL1, TREM2, ABCA7, ATP8B4 and ABCA1 genes. The association signal of the ADAM10 gene was not exome-wide significant, presumably because the Stage-2 dataset encompassed too few prioritized variants in this gene.
Randomization	Data quality control was performed across all data, irrespective of case and control status.
Blinding	To perform burden analyses, the analysts were not blinded to the status of the individuals because this requires knowing case and control status.

Reporting for specific materials, systems and methods

We require information from authors about some types of materials, experimental systems and methods used in many studies. Here, indicate whether each material, system or method listed is relevant to your study. If you are not sure if a list item applies to your research, read the appropriate section before selecting a response.

Materials & experimental systems

n/a	Involved in the study
☒	☐ Antibodies
☒	☐ Eukaryotic cell lines
☒	☐ Palaeontology and archaeology
☒	☐ Animals and other organisms
☐	☒ Human research participants
☒	☐ Clinical data
☒	☐ Dual use research of concern

Methods

n/a	Involved in the study
☒	☐ ChIP-seq
☒	☐ Flow cytometry
☒	☐ MRI-based neuroimaging

Human research participants

Policy information about studies involving human research participants

Population characteristics	We used multiple independent sets of participants in this study. We adjusted the analysis for principal components. Sample sizes, age and gender characteristics for our sample can be found per cohort and in Supplementary Table 1 of the Supplementary Note, and in the cohort descriptions in Section 1.1 of the Supplementary Note.
Recruitment	Participants from case-control studies were primarily recruited from clinics, nursing homes, disease registries, and hospitals, with controls being drawn from various ongoing studies and screened to exclude dementia/cognitive decline (Please see recruitment procedures in the cohort descriptions in section 1.1 of the Supplementary Note). Cases were recruited according to clinical diagnosis and defined as probable AD cases with a potential risk of misdiagnosis (estimated between 10 and 20% in the literature). Controls included in the study were free of cognitive decline but a large part of them did not have any follow-up wit the possibility that they developed dementia years later.
Ethics oversight	Written informed consent was obtained from study participants or, for those with substantial cognitive impairment, from a caregiver, legal guardian, or other proxy, and the study protocols for all populations were reviewed and approved by the appropriate local Institutional Review Boards (see description of the samples in the Supplementary Note).

Note that full information on the approval of the study protocol must also be provided in the manuscript.
